# An Audit of Junior Medical Seclusion Review Documentation in the Adult Psychiatric Intensive Care Unit (PICU) setting

**DOI:** 10.1192/bjo.2024.630

**Published:** 2024-08-01

**Authors:** Anju Sharma, Afshan Khawaja

**Affiliations:** Greater Manchester Mental Health, Manchester, United Kingdom

## Abstract

**Aims:**

Seclusion facilities are frequently used in adult psychiatric intensive care units (PICUs). Seclusion refers to the supervision of a service user in a secure area.

Aim: To evaluate whether trust standards for seclusion review assessments at Park House Hospital were being met.

**Objectives:**

To measure the quality of junior medical review documentation to determine whether reviews of physical health, risk, medication, and mental state exams (MSEs) were included. The time frames in which reviews were being undertaken and the rationale for seclusion were considered.

**Methods:**

A retrospective audit of notes on the electronic patient information system was completed. Those included were patients secluded between May 2022–October 2022. The majority of seclusions occur on the male PICU, or 136 suite. Eligible patients were identified following consultation with the business intelligence team within Greater Manchester Mental Health (GMMH). For those who had multiple periods of seclusion, the first episode of seclusion was audited. Data were obtained from the last recorded junior review prior to the seclusion episode being terminated. Progress notes and the internal MDT review documents were searched. This was compared against the local trust seclusion policy.

**Results:**

20 patients were included in the audit. The majority had a diagnosis of either paranoid schizophrenia (40%) or schizoaffective disorder (25%). 95% of seclusion reviews had a clearly documented initiation time and rationale for seclusion. Physical health considerations were documented in 75% of reviews. 50% of junior reviews documented an assessment of risk to others, compared with 5% of reviews with documented review of risk to self. Half of all reviews had evidence of a MSE and medication review, including the use of rapid tranquilisation (RT). Of the reviews eligible for initial medical review within 60 minutes, this was completed in 44% of cases.

**Conclusion:**

Junior medical reviews have consistently documented the rationale for seclusion and physical health reviews. Areas for development include clear documentation of MSE however documentation may be limited due to time constraint, lack of engagement from the patient or if patients are asleep. The policy since time of audit has changed to reflect this, where consideration must now be given to “overall psychiatric health”. It was found that risk to self largely remains undocumented, despite trust policy. There is evidence to suggest risk to self may increase during a period of seclusion. Another area of development includes medical review documentation to specifically comment on use of RT.

